# Medical licensing examinations in both Sweden and the US favor pharmacology over lifestyle

**DOI:** 10.1016/j.pmedr.2021.101453

**Published:** 2021-06-17

**Authors:** B. Krachler, L. Jerdén, H. Tönnesen, C. Lindén

**Affiliations:** a Department of Public Health and Clinical Medicine, Sustainable Health, Umeå University, Umeå, Sweden; b Center for Clinical Research Dalarna-Uppsala University, Falun, Sweden; c School of Education, Health and Social Studies, Dalarna University, Falun, Sweden; d Clinical Health Promotion Centre, WHO-CC, Region Skåne and Department of Health Sciences, Faculty of Medicine, Lund University, Sweden; e Department of Clinical Sciences, Ophthalmology, Umeå University, Umeå, Sweden

**Keywords:** Medical education, Assessment, Step 3, Graduate, Living habits, Health behavior

## Abstract

Low priority of disease prevention and health promotion in medical education may contribute to lack of lifestyle-counseling in clinical practice. Pharmacology-related knowledge is valued 5 times higher compared to lifestyle-related knowledge in examinations on noncommunicable diseases (NCDs) in undergraduate medical education in Sweden. This study aims to establish (i) whether medical licensing examinations are biased to favor pharmacology- over lifestyle-related knowledge and (ii) whether such a bias is present in both Sweden and the US.

We identified 204 NCD-related questions from previous Swedish licensing examinations, and 77 cases from a U.S. question bank commonly used to prepare for the United States Medical Licensing Examination® (USMLE®) Step 3. With the help of expected correct answers, we determined distribution of points attainable for knowledge in the respective category (lifestyle / pharmacology / other) for 5 major NCDs: coronary heart disease (CHD), chronic obstructive pulmonary disease (COPD), diabetes, hypertension, and stroke.

The percentage of points attainable for lifestyle-related knowledge was 6.7 (95% CI 4.1–9.3) in Sweden and 4.6 (95%CI 0.0–9.1) in the U.S. The respective percentages for pharmacology-related knowledge were 32.6 (95% CI 26.3–38.8) and 44.5 (95% CI 33.2–55.8) percent. The pharmacology vs. lifestyle-quotas were 4.9 in Sweden and 9.8 in the U.S. Likelihoods of equal emphasis on lifestyle and pharmacology in NCDs was < 0.001 in both countries.

There is a marked preference for pharmacology over lifestyle in medical licensing examinations in both Sweden and the U.S. Newly qualified doctors may be inadequately prepared to address preventable causes of NCDs.

## Highlights

• Lifestyle is a causative factor in most non-communicable diseases.
• Medical licensing examinations emphasize pharmacology over lifestyle.
• Future doctors in Sweden and US may not be equipped to address lifestyle-factors.

## 1 Introduction

According to the World Health Organization, noncommunicable diseases (NCDs) account for 41 million deaths each year, equivalent to 71% of all deaths globally (World Health Organization, 2018). There is consensus that modifiable behaviors, such as food habits, physical activity, smoking and alcohol use, have a major impact on NCDs like cardiovascular disease, chronic obstructive pulmonary disease and diabetes (G B D Disease Injury Incidence Prevalence Collaborators, 2018). More than 30 years ago, a *meta*-analysis convincingly demonstrated the effects of physicianś advice to their patients on smoking cessation (Kottke, 1988). Eventually, counseling and/or advice from health care personnel have been shown to be effective tools to counteract harmful alcohol consumption (Kaner et al., 2018), insufficient physical activity (Sanchez et al., 2015, Pavey et al., 2013) and unhealthy eating habits (Ball, 2015, Rees, 2013). However, there are reports indicating substantial shortcomings in the handling of unhealthy lifestyle in health care (Osborn et al., 2014). Large differences in the provision of preventive services in primary care have also been reported, both between European countries (Brotons et al., 2005), and between the United States and Sweden (Jerdén et al., 2018).

One possible reason for difficulties to implement lifestyle counseling in health care might be a low priority of lifestyle issues in medical education. Earlier results indicate that pharmacology-related knowledge renders five times as many points compared to lifestyle-related knowledge in examinations on NCDs in undergraduate medical education in Sweden (Krachler et al., 2019).

In Sweden, medical education consists of 5½ years, followed by a compulsory 2-year internship. Thereafter, an exam must be passed before the medical license is provided which in turn is prerequisite for residency training. In the U.S., 4 years of medical education are followed by residency training, after the first year of which the United States Medical Licensing Examination® (USMLE®) step 3 must be passed for licensing. Part of the difference in total duration of medical training is explained by a compulsory pre-medical education at college level in the U.S.

The aim of the present study is to establish (i) whether medical licensing examinations are biased to favor pharmacology- over lifestyle-related knowledge and (ii) whether such a bias is present in both Sweden and the US. Hence, the null-hypotheses to be tested are: examinations after postgraduate clinical training put an equal emphasis on lifestyle-factors and pharmacology in the context of NCDs in both Sweden and the United States.

## 2 Methods

From the Swedish national database of previous licensing examinations (Karolinska Institutet, 2020) we retrieved all 34 examinations held between 2010 and fall 2018.^1^ As we were denied access to the USMLE®-database^2^ we chose a commercial question bank (UWorld for USMLE® step 3 (UWorld for USMLE step 3., 2020), commonly used by students to prepare for USMLE® exams (Bhatnagar et al., 2019, Seal et al., 2020) and previously described as most representative of questions seen on USMLE® (Andyryka, 2014). We confined ourselves to the following NCDs: coronary heart disease (CHD), chronic obstructive pulmonary disease (COPD), diabetes, hypertension, and stroke. Questions regarding prevention of thromboembolism in atrial fibrillation were classified as stroke related. We identified 204 questions related to these NCDs in the Swedish, and 77 in the U.S. question bank.

### 2.1 Analysis of examinations

Two authors (BK, CL) read all examinations and identified questions concerning lifestyle-related disease. We discriminated between three categories of knowledge: Lifestyle-related, pharmacology-related, and other. The latter category includes knowledge about pathophysiology, clinical examination, investigation, interventions other than lifestyle- or pharmacological, differential diagnoses, ethical considerations, etc. All questions concerning pharmacological treatment of the five NCDs were categorized as pharmacology related. Questions regarding lifestyle habits or health behavior change in the context of one of the five above-stated NCDs were categorized as lifestyle-related. Discrimination between respective categories was solely based on clinical experience and pedagogical expertise of raters. A third author (LJ) independently categorized all lifestyle-related questions with respect to obtainable points in the respective categories for right answers as well as individual lifestyle components in the background information. Conflicts of opinion regarding category and/or lifestyle components were resolved by discussion to agreement. A summary of principles for assessment is given in the Appendix. These principles were applied, and if necessary appended, whenever disagreements occurred.

**Fig. 1:**
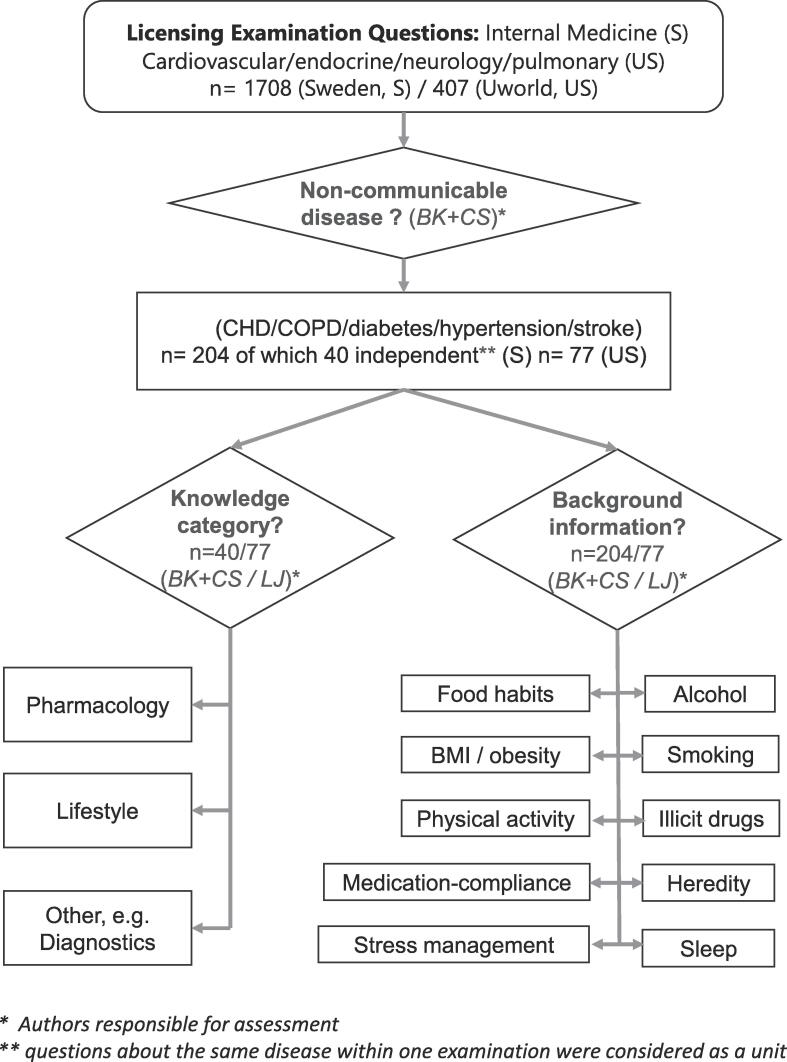

A flow-chart of the assessment process is given in Fig. 1.

With the help of expected correct answers, we determined distribution of points attainable for knowledge in the respective category (lifestyle / pharmacology / other). For the few hybrids, i e questions that contained elements of both lifestyle and pharmacology, proportions of available points within the respective category were counted e.g., a 2-point multiple essay questions with one expected answer in each category rendered 1 point for both lifestyle and pharmacology. A multiple-choice question with 1 correct answer and 2 distractors within pharmacology and 2 further distractors in lifestyle would have rendered 0,6 points for pharmacology and 0,4 points for lifestyle (hypothetical example as both correct answer and distractors were as a rule in the same category).

To further study the relative importance given to individual components of lifestyle we also categorized information given in vignettes regarding medication compliance, use of illicit drugs, alcohol, smoking, food habits, sleep habits, physical activity, stress management and - as a surrogate measure of energy balance - the presence or absence of obesity. As “genes vs. lifestyle” is a common topic in NCDs we also registered whether information regarding heredity was provided.

### 2.2 Handling of differences between Swedish examinations and the US-question-database

Most of the studied Swedish licensing examinations consisted of multiple essay questions with incremental provision of background information in the vignette for each subsequent question. For the purpose of testing the hypothesis of equal weight given to pharmacology-and lifestyle-related knowledge (p-values in Table 1) we considered all questions regarding a specific NCD within the same Swedish examination as one unit. Such, the 204 NCD-related individual questions resulted in 40 units (Table 1). To establish the relative weight of pharmacology and lifestyle (Fig. 1) and the nature of background information regarding lifestyle-factors (Table 2) the separate sub-questions in a multiple essay-question were considered as independent i.e., the sum total of background information provided so far was recorded separately for each sub-question. The UWorld database contained only multiple-choice questions. Each question from the UWorld database was given similar weight and considered as an independent unit, even if 2 questions shared the same vignette (as was the case for 12 out of 77 NCD-related questions).

**Table 1:** Likelihood of equal weight for lifestyle- vs. pharmacology-related knowledge. Comparison of points attainable at medical licensing examinations in Sweden and the U.S.-UWorld® question-database.

**US** (questions from database)									**Sweden** (independent questions at examinations)				
n^a^=		Pharmacology	Lifestyle	Other	p^a^_Lifestyle=Pharmacology_				n^a^=	Pharmacology	Lifestyle	Other	p^a^_Lifestyle=Pharmacology_
27		10	1	16	0,012		**CHD**		7	2	2	3	n.s.
7		1	2	4	n.s.		**COPD**		8	3	2	3	n.s.
25		16	0	9	< 0,001		**Diabetes**		10	7	1	2	n.s.
4		2	1	1	n.s.		**Hypertension**		7	6	1	0	n.s.
14		5	0	9	n.s.		**Stroke**		8	8	0	0	0,008
77		34	4	39	< 0,001		**all 5 NCD: s**		40	26	6	8	0,001

a Units for sign-test are individual questions from the UWorld database for the U.S. and all questions regarding a single NCD at a particular exam for Sweden.

**Table 2:** Background information on lifestyle factors given in vignettes of cases of NCDs in the U.S. (U-World) and Sweden (medical licensing examinations). CHD = Coronary Heart Disease, COPD = Chronic Obstructive Pulmonary Disease.

	Diabetes		CHD		Stroke		Hypertension		COPD		average	
	U.S.	Swe	U.S.	Swe	U.S.	Swe	U.S.	Swe	U.S.	Swe	U.S.	Swe
n=	25	32	27	36	14	56	4	24	7	56	77	204
medication-compliance	36%	3%	7%	0%	0%	0%	50%	25%	0%	13%	17%	7%
illicit drugs	36%	0%	22%	0%	7%	0%	75%	0%	29%	0%	27%	0%
alcohol	56%	41%	48%	3%	29%	23%	100%	42%	86%	13%	53%	22%
smoking	60%	31%	63%	92%	36%	52%	100%	63%	86%	77%	61%	64%
heredity	32%	31%	48%	11%	14%	2%	25%	38%	57%	14%	36%	16%
sleep	0%	16%	7%	0%	0%	2%	0%	29%	14%	0%	4%	6%
BMI/obesity	44%	81%	41%	3%	14%	50%	50%	67%	14%	46%	35%	48%
food habits	28%	19%	0%	3%	0%	2%	0%	0%	0%	4%	9%	5%
physical activity	28%	25%	11%	39%	0%	2%	0%	42%	14%	14%	14%	20%
stress management	0%	3%	4%	3%	0%	2%	0%	13%	0%	0%	1%	3%
	32%	25%	25%	15%	10%	13%	40%	32%	30%	18%	26%	19%

### 2.3 Statistical analyses

We used the Statistical Analysis System (SAS for Windows, version 9.4, SAS Institute, Carry, NC 27513, USA) for all statistical evaluations.

To determine the likelihood of equal weight for lifestyle- and pharmacology-related knowledge, we calculated the difference between obtainable points in respective category (lifestyle vs pharmacology) for questions regarding NCDs in Sweden and the U.S. We used the sign test in the PROC UNIVARIATE procedure to obtain respective P-values for the hypothesis of equal or higher number of obtainable points for LM-knowledge compared to pharmacology-related knowledge.

To assess the relative importance of lifestyle and pharmacology we noted the percentage of obtainable points for all questions regarding the 5 NCDs in both Sweden and the U.S. Comparison of means aggregated over all 5 NCDs was performed by T-test with PROC MEANS procedure.

### 2.4 Ethical considerations

This study did not involve human participants and is therefore exempt from formal ethics review.

## 3 Results

Except for COPD, questions concerning NCDs favored pharmacology-related knowledge. The percentage of points attainable for lifestyle-related knowledge was 6.7 (95% CI 4.1–9.3) in Sweden and 4.6 (95%CI 0.0–9.1) in the U.S. The respective percentages for pharmacology-related knowledge were 32.6 (95% CI 26.3–38.8) and 44.5 (95% CI 33.2–55.8) percent. The pharmacology vs. lifestyle-quotas were 4.9 in Sweden and 9.8 in the U.S.

Likelihoods of equal emphasis on lifestyle and pharmacology in NCDs is given in Table 1.

**Fig. 2:**
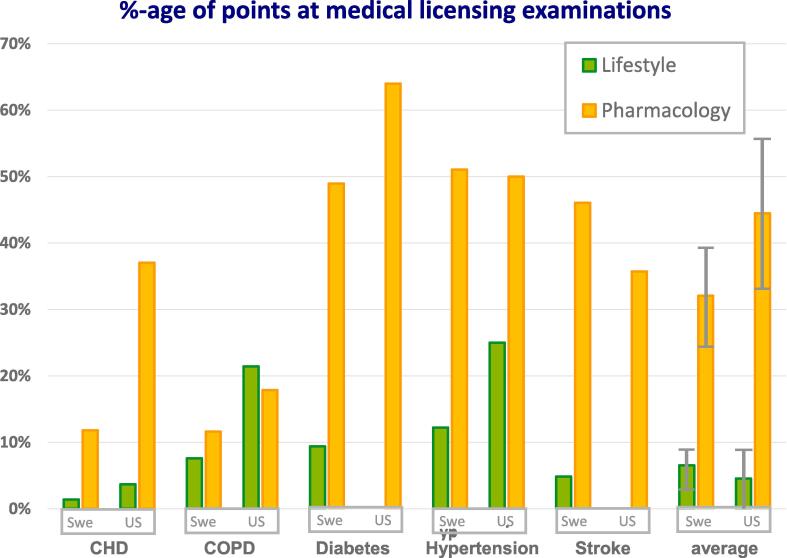

Distribution of points awarded in the respective categories is given in Fig. 2.

An overview of information on individual lifestyle components provided in vignettes is given in Table 2.

Stress-management, sleep and food habits are mentioned in < 10% of the vignettes in both countries, use of illicit drugs and medication compliance receive low attention in Sweden. Smoking is the only lifestyle component that is mentioned in more than half of the vignettes in both countries.

## 4 Discussion

The null-hypothesis of an equal emphasis on lifestyle- and pharmacology-related knowledge in licensing examinations could not be confirmed, as there were substantial differences in both countries when questions about all five NCDs were considered together. The multiple at which lifestyle-related knowledge was discounted in postgraduate examinations in Sweden is almost identical with that which was previously found in undergraduate examinations on national level (4,8 vs 5,5 times lower priority) (Sanchez et al., 2015). Content of the UWorld database discounted lifestyle-related knowledge at higher rate, similar to undergraduate examinations at the most prestigious Swedish medical school, Karolinska Institutet (9,8 vs 9,3 times lower priority). Except for smoking (both countries), alcohol (U.S) and obesity (Sweden), relatively little lifestyle-related information is provided in vignettes.

How various *forms* of assessment influence students’ learning and/or performance has been studied extensively (Scott, 2020, Struyven et al., 2006). The question whether assessment-*content* drives learning has received less attention, probably because it is considered as self-evident. We do not have a definite suggestion, on what should be an adequate proportion between lifestyle- and pharmacology-related knowledge in medical education. There is potential for harm in suboptimal pharmacological treatment but there is also potential for harm in failing to address lifestyle as both a causative and curative factor: Especially concerning CHD, diabetes and stroke, questions about lifestyle are absent, or priority in terms of available points is extremely low. As lifestyle-factors have the potential to cut all-cause mortality by half (Zhang et al., 2021), and lifestyle interventions are a cornerstone in long-term management of these diseases (Collet et al., 2021, Williams et al., 2020, American Diabetes, 2021), the marked preference for pharmacology over lifestyle found in this study appears inappropriate.

Thus, newly qualified doctors may not be adequately prepared for the spectrum of lifestyle-related disease in the 2020s. Moreover, there are widespread patient expectations of lifestyle counseling, as shown by the EUROPREVIEW study, conducted in 22 European countries: half of patients with smoking, unhealthy eating habits or lack of physical activity wanted their general practitioners to offer advice about lifestyle habits (Brotons et al., 2012). To meet these needs, medical education must give higher priority to knowledge about the impact of lifestyle on NCDs.

As there is ample evidence of the eminent importance of addressing lifestyle in both treatment and prevention of NCDs the bias towards pharmacological treatment found in our study may reflect medical culture (Scott, 2020) i.e. the existence of an informal prestige hierarchy. This hierarchy has been studied in terms of specialty and diseases (Creed et al., 2010, Album et al., 2017), but it may actually be the required form of intervention that defines the prestige of a disease and pharmacological interventions may score higher than efforts to change living habits (Haldar et al., 2016). Interestingly, information regarding compliance with prescribed medication is given as sparingly as information regarding other lifestyle components. With a view to the fact that only 50% of patients are taking their medications as prescribed (Brown et al., 2016), there is a lack of consequence even in the preference for rewarding knowledge concerning pharmacological treatment of lifestyle related diseases.

Comparisons of medical education across universities and countries (Zavlin et al., 2017, Wilkinson et al., 2014, Wilkinson et al., 2015) as well as different approaches to curriculum design (Miles et al., 2017, Berkenbosch et al., 2013, Lucardie, 2017) and forms of assessment (Haist et al., 2017, Pearce et al., 2015) have been conducted earlier. Likewise, the representation of selected disciplines and diseases in licensing examinations have been investigated (Fishman et al., 2018, Hark et al., 19971997, Kushner et al., 2017). However, we are not aware of studies of the relative importance of two domains within the context of licensing examinations.

The fact that we studied examination-content rather than medical school’s curriculum descriptions is a major strength of our study. It covers every single written Swedish licensing examination given between 2010 and 2018. Compared to our previous survey of undergraduate examinations (Krachler et al., 2019), the current one covers even all general practice-questions. The UWorld database does not contain questions actually given at licensing examinations which may be seen as a limitation. However, UWorld questions have been deemed as most closely resembling USMLE® (Andyryka, 2014) and UWorld scores are good predictors of USMLE Step 1 scores (Seal et al., 2020, Giordano et al., 2016). Moreover, the fact that original USMLE® questions are kept secret limits their educational impact and makes comparison of UWorld with the equally open Swedish questions relevant.

The current study has several limitations: The absence of a formalized a-priori protocol and the small number of independent assessors (BK + CL / LJ) may raise questions about the reliability of the results. BK and LJ are experienced clinicians in internal medicine and general practice, respectively. The 4 co-authors have a combined experience of several decades of teaching in higher medical education. Principles for assessment are provided in Appendix 1. As internal medicine, the main domain of NCDs, only comprises 25% of licensing examination content in Sweden and even less in the U.S., the total number of questions is comparatively small. Thus, the statistical power to detect differences is limited. As both types of questions (multiple essay in Sweden, multiple choice in UWorld) and settings (actual examination in Sweden, question bank for preparation in US) differ, direct comparisons between the countries are difficult. Also, practical examinations, held and evaluated separately from the nation-wide written licensing examinations, are not covered by the current survey.

## 5 Conclusions

Our results indicate that both Swedish and U.S. medical licensing examinations put a marked emphasis on pharmacology in the management of lifestyle related NCDs. This may in turn mold future medical doctors to focus on pharmacological interventions and give lower priority to health promotion.

## Disclosure

This research did not receive any specific grant from funding agencies in the public, commercial, or not-for-profit sectors.

## CRediT authorship contribution statement

**B. Krachler:** Conceptualization, Methodology, Formal analysis, Writing - original draft. **L. Jerdén:** Methodology, Formal analysis, Writing - review & editing. **H. Tönnesen:** Methodology, Writing - review & editing. **C. Lindén:** Conceptualization, Methodology, Formal analysis, Writing - review & editing.

## Footnotes

1 At the time of data collection, the public database contained examinations starting 2010, since then examinations for 2010–2012 have been removed.

2 At the time of data collection for this publication the USMLE® program was reviewing its policy to engage interest groups in future review activities.
