# Supplementary material for: Medical licensing examinations in both Sweden and the US favor pharmacology over lifestyle
Source: Prev Med Rep. 2021 Jun 17;23:101453. doi: 10.1016/j.pmedr.2021.101453 (PMC8227799; doi:10.1016/j.pmedr.2021.101453)
Supplement: Supplementary data 1 [file mmc1.docx]

Licensing examination review

Principles for categorizing examination questions as non-communicable diseases, knowledge categories (pharmacology/lifestyle/other) and lifestyle-related background information.

The basic idea is to assess the points available in the domain of lifestyle-related diseases for each examination question. Based on this number we further assess the proportion of points that can be earned by

1. knowledge about either lifestyle as a risk factor or lifestyle interventions as a means of treatment
2. knowledge about pharmacological treatment
3. other types of knowledge e.g., diagnostic algorithms.

In a further step we aim to assess information on living habits given in the vignette as such information also may reflect the relative importance given to living habits in an examination context.

## Inclusion criteria: Lifestyle-related non-communicable diseases

1. Type 2 diabetes, but also type 1 diabetes as living habits (including compliance with medication) are the most important prognostic factor.
2. Coronary heart disease
3. Stroke (both thromboembolic and hemorrhagic, but not subarachnoid hemorrhage)
4. Hypertension
5. (Sleep apnea – removed later due to lack of cases precluding comparisons)
6. Chronic obstructive pulmonary disease
7. (Alcohol-related liver disease – removed later due to lack of cases precluding comparisons)
8. (Wernicke-Korsakoff syndrome – removed later due to lack of cases precluding comparisons)
9. (Drug intoxication – removed later due to inconsistencies as causative treatment is often in the domain of psychiatry whereas symptomatic treatment is part of internal medicine)
10. (Generic questions regarding living habits – removed later due to lack of cases precluding

comparisons)

- All questions regarding pathogenesis, diagnosis and treatment of above-mentioned conditions contribute to the total sum of points available for lifestyle-related NCDs.
- Other conditions are *not* regarded as lifestyle-related, even if they are complications of lifestyle related diseases (e.g., heart failure after cardiac infarction, infected diabetic foot ulcers).

## **Knowledge categories (pharmacology/lifestyle/other):**

- Questions on complications of lifestyle-treatments (e.g., palpitations after having been advised to exercise more for reduction of hypertension) contribute to lifestyle-points, complications of pharmacological treatments (e.g., gastrointestinal problems after prescription of metformin for type 2 diabetes) contribute to pharmacology.
- Questions regarding lifestyle-related NCDs are regarded as lifestyle-related, even if the specific case turns out to be due to a less common non-lifestyle condition (e.g., hypertension: secondary hypertension, malignant hypertension; diabetes: LADA, secondary diabetes). This enables us to capture a possible bias towards over-emphasis on non-lifestyle causes to conditions that are generally lifestyle-related.
- Questions regarding (patho-)physiological and biochemical mechanisms contribute to the respective categories (e.g., pharmacology: mechanism for slow-release insulin; lifestyle: mechanisms for increased insulin sensitivity with increased physical activity).
- Questions regarding the role of lifestyle as a risk factor, as a means of treatment or as a means of prevention all contribute to lifestyle.
- Questions where lifestyle is one of several alternatives contribute to lifestyle in proportion with the number of alternatives. (E.g., If the maximum points of the question are 2 points and a fully correct answer contains 2 pharmacology-related parts, one lifestyle-related part and one part concerning something else, it renders 0,5 p lifestyle, 1 p pharmacology, 0,5 p other).
- Questions on rules regarding social benefits in lifestyle related diseases contribute to “other”, not lifestyle (e.g., sick-leave during period of instable blood sugar level).
- Questions regarding ability to drive a vehicle after stroke contribute to “other”, not lifestyle.
- Pharmacological treatment of lifestyle-factor renders equal points (50% each) for both lifestyle and pharmacology (e.g., pharmacological intervention for smoking cessation in patients with hypertension).

## **Background information on lifestyle factors:**

- Information on activity-related chest-pain is only regarded as background information on physical activity if information on the patients’ habitual activity level is provided (e.g., chest pain when walking = NO; chest pain when she takes her daily 30 min brisk-walk = YES).
- If information on heredity is provided in the vignette it is counted as positive, even if the specific disease is not mentioned (e.g., “father died early of heart attack” when the disease in question is stroke).
- Statements in the vignette regarding living habits are only counted as lifestyle-related if direct or indirect information regarding the patients living habits is provided (e.g., “the patients received advice regarding diet and exercise” = NO; “food habits have not changed” = YES; “she is fond of bakery-products” =YES; “the patient got advice to increase his current activity level” =YES).
- Even general information on level of stress is regarded as background information on stress management, even if no specific stressor is mentioned (e.g., “has a high level of stress” =YES).
